# π-SeqOmics: A Sequential Workflow for Genomic, Transcriptomic, Proteomic, and Phosphoproteomic Profiling From Biopsy-Scale Samples

**DOI:** 10.1016/j.mcpro.2026.101596

**Published:** 2026-05-28

**Authors:** Shuyi Feng, Xuehui Deng, Ying Xu, Baoyi Qin, Chuanxi Huang, Qingjing Chen, Fuchu He, Dongxue Wang

**Affiliations:** 1 State Key Laboratory of Medical Proteomics, National Center for Protein Sciences (Beijing), Research Unit of Proteomics-driven Cancer Precision Medicine (Chinese Academy of Medical Sciences), Beijing, China; 2 International Academy of Phronesis Medicine, Guangzhou, Guangdong, China; 3 Department of Chemistry, School of Science, Southern University of Science and Technology, Shenzhen, China; 4 Nanfang Hospital, Southern Medical University, Guangzhou, China; 5 Beijing Proteome Research Center, Beijing, China

**Keywords:** genome, transcriptome, proteome, phosphoproteome, integrated workflow

## Abstract

Comprehensive multiomics profiling from a single limited specimen remains challenging, as current extraction methods are difficult to standardize and often compromise balanced recovery of nucleic acids and proteins. Here, we developed π-SeqOmics, a phenol-free workflow established through systematic benchmarking for the sequential isolation of DNA, RNA, and proteins from a single specimen, enabling integrated genomic, transcriptomic, proteomic, and phosphoproteomic analyses. Compared with conventional extraction and in-solution digestion workflows, π-SeqOmics achieves comparable proteomic and phosphoproteomic depth and reproducibility while preserving high-quality DNA and RNA for sequencing. The workflow performed robustly across a wide input range (5 × 10^6^ – 1 × 10^5^ cells) and multiple mouse tissues, consistently identifying over 8000 proteins with strong quantitative reproducibility (r > 0.95). Together, these results establish π-SeqOmics as a standardized, practical, and cost-effective multiomics sample preparation workflow for limited-input and biopsy-scale specimens, providing a robust foundation for systems biology and translational research.

## Highlights

• π-SeqOmics enables sequential isolation of DNA, RNA, and proteins from the same specimen.
• Provides a phenol-free, standardized workflow using simple and cost-effective reagents.
• Supports robust and reproducible multiomics profiling from limited-input samples.

## Graphical Abstract

**Figure:**
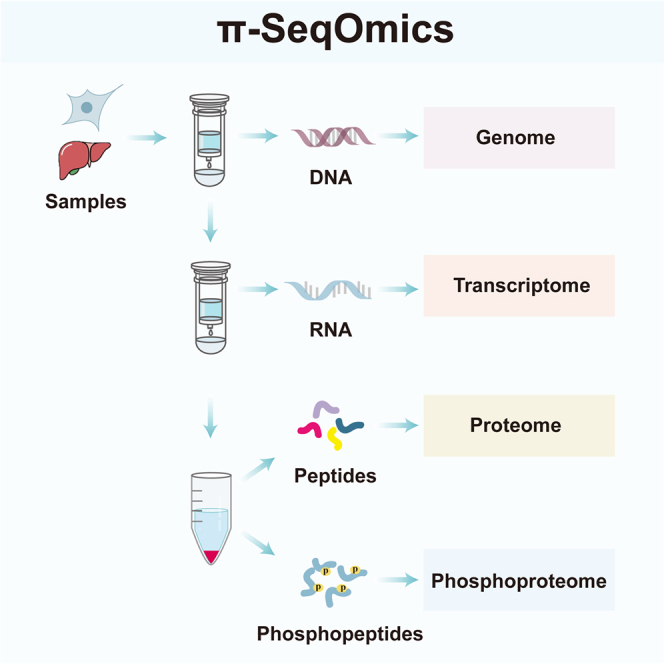

Systems biology and precision medicine increasingly rely on multiomics approaches that capture multiple molecular layers within the same sample (1, 2, 3). By integrating genomics, transcriptomics, proteomics, and posttranslational modifications such as phosphorylation, these strategies provide a comprehensive view of disease mechanisms, enable biomarker discovery, and guide therapeutic interventions (3, 4, 5, 6, 7, 8, 9, 10, 11, 12). The genome provides a static blueprint, the transcriptome reflects dynamic expression, and the proteome—further shaped by posttranslational modifications—acts as the cellular effector (13, 14). Obtaining these layers from the same specimen reduces heterogeneity introduced by sample splitting and improves the reliability of downstream analyses (15). Recent advances in proteogenomics have highlighted the value of analyzing multiple molecular layers from the same specimen, but routine application remains constrained by the limited material typically available from clinical biopsies or rare cell populations (16).

Several approaches have been developed for single-sample multiomics extraction, but important limitations remain. Phenol/chloroform-based protocols such as TRIzol and RNA-Bee enable sequential recovery of DNA, RNA, and proteins and have been extended to proteomic and phosphoproteomic analyses (17, 18, 19). Satpathy *et al*. (20) applied this concept to biopsy-scale proteogenomics using serial sectioning of optimal cutting temperature compound-embedded core biopsies, phenol-containing RNA extraction, and recovery of denatured proteins from the DNA and RNA flow-throughs for proteomic and phosphoproteomic analysis. Integral-Omics further extended phenol/chloroform-based workflows to the sequential extraction of metabolites, lipids, genomic DNA, total RNA, proteins, and phosphopeptides from limited material (21). However, these workflows remained multistep and technically demanding, making standardization challenging. Phenol-free approaches have also been explored. For example, Mundt *et al*. (22) demonstrated the feasibility of proteomic and phosphoproteomic analysis from protein-containing fractions generated during commercial DNA/RNA extraction, but this work primarily focused on feasibility and protein recovery optimization rather than systematic benchmarking of extraction workflows or establishment of a standardized end-to-end protocol. Other methods, such as ProMTag, use a reversible protein-tagging scheme together with nucleic acid precipitation and selective solubilization to generate nucleic acid fractions and MS-compatible proteins from the same sample, but rely on specialized reagents and have so far been validated only in limited systems (23, 24). Collectively, these studies highlight both the promise of same-specimen multiomics and the continuing need for practical, standardized, and phenol-free workflows that preserve nucleic acid quality while remaining compatible with deep proteomic and phosphoproteomic analysis (25, 26, 27).

Here, we present π-SeqOmics, a sequential, phenol-free workflow that combines spin-column–based DNA/RNA extraction with ZnCl2 precipitation-assisted proteomic sample preparation. Building on systematic comparisons of nucleic acid–centric protocols and precipitation strategies, this design yields high-quality genomic DNA, total RNA, and proteins while avoiding phenol carryover and the biases introduced by sample splitting. The workflow supports integrated genomic, transcriptomic, proteomic, and phosphoproteomic analyses from the same specimen. We benchmarked π-SeqOmics against the in-solution digestion (ISD) workflow in Human embryonic kidney (HEK) 293T cells, assessed sensitivity using cell-number titrations (≥1 × 10^5^ cells), and evaluated robustness across diverse mouse tissues, including brain, heart, lung, kidney, and rectum. Recovered DNA and RNA were also assessed for quantity and integrity to verify their suitability for downstream genomic and transcriptomic analyses. Together, these results establish π-SeqOmics as a standardized, practical, and cost-effective solution for biopsy-scale multiomics studies.

## Experimental Procedures

### Cell Culture

HEK 293T cells were cultured in Dulbecco's modified Eagle's medium (Gibco) supplemented with 10% fetal bovine serum, and 1% penicillin/streptomycin (Gibco) in an incubator with 5% CO_2_ at 37 °C. Upon reaching 80% confluence, HEK 293T cells were harvested using 0.05% (v/v) trypsin-EDTA solution and collected in 15 ml Falcon tubes. Subsequently, HEK 293T cells were washed three times with PBS and frozen at −80 °C until further use.

### Mouse Tissue Collection

Mice used in this study were maintained at the Beijing Proteome Research Center in accordance with institutionally approved conditions. All experimental procedures were conducted in accordance with the National Institutes of Health Guide for the Care and Use of Laboratory Animals and approved by the Institutional Animal Care and Use Committee of the Beijing Proteome Research Center (Approval No. NCPSB-20230524-31MT). Mouse tissues in these experiments were healthy and collected from three healthy 8-week-old C57BL/6 mice.

### Protein Extraction from Cells and Tissues

HEK 293T cells and mouse heart tissues were lysed in sodium deoxycholate (SDC) buffer (1% SDC, 100 mM Tris–HCl, pH 8.5) and boiled at 95 °C for 5 min to denature proteins and inactivate proteases. HEK 293T cells were then sonicated for 10 min on a Qsonica Q800R3 sonicator (3 s on/3 s off, 85% amplitude) to shear nucleic acids and enhance protein solubilization. Mouse hearts were homogenized on an OMNI Bead Ruptor 24 Elite (7.1 m/s, three cycles, 10 s on, 10 s off) and subsequently sonicated (10 s on/10 s off, 85% amplitude). Insoluble debris was removed by centrifugation at 14,000 × *g* for 10 min, and clarified lysates were stored at −80 °C until further use. Protein concentrations were measured using the Pierce bicinchoninic acid Protein Assay Kit (Thermo Fisher Scientific).

### Multiomics Extraction via TRIzol

HEK 293T cells were lysed in TRIzol Reagent, and RNA, DNA, and proteins were sequentially isolated by standard phase separation. RNA was recovered from the aqueous phase, DNA was precipitated from the interphase and organic phase, and proteins were collected from the remaining supernatant. To compare precipitation strategies for protein recovery, proteins were processed using either acetone precipitation or ZnCl2 precipitation-assisted sample preparation (ZASP).

The acetone precipitation method was adapted from Pena (25) with some modifications, a 4-fold volume of ice-cold acetone was added to the protein supernatant, followed by incubation at −20 °C for 30 min (or overnight) and centrifugation at 19,000 × *g* for 10 min at 4 °C. The ZASP protocol was adopted from Shao (28), an equal volume of ZASP buffer (99.9% methanol, 0.1% formic acid [FA], 200 mM ZnCl_2_) was added, incubated for 10 min at room temperature, and centrifuged at 19,000 × *g* for 10 min at 4 °C; the resulting pellet was washed once with freshly prepared methanol containing 0.1% FA. Protein pellets obtained from either method were resuspended in SDC buffer and subjected to ISD.

### Multiomics Extraction via Column-Based DNA/RNA Coextraction

DNA and RNA were extracted using the DNA/RNA coextraction (DR) Kit (TIANGEN) according to the manufacturer’s protocol. After RNA isolation, the flow-through from the RNase-Free column CR3 was retained and stored at −20 °C for subsequent protein recovery. Proteins were precipitated from this fraction using either acetone or ZASP precipitation, as described in the TRIzol protocol, and the resulting pellets were resuspended in SDC buffer for ISD.

### Multiomics Extraction via Column-Based DNA/RNA/Protein Coextraction

DNA and RNA were extracted using the DNA/RNA/Protein coextraction (DRP) Kit (TIANGEN) according to the manufacturer’s protocol. After RNA isolation, the flow-through from the RNase-Free column CR3 was retained and stored at −20 °C for subsequent protein recovery. Proteins were precipitated from this fraction using acetone precipitation, ZASP precipitation, or the kit-provided PR reagent, the latter serving as a positive control for protein recovery efficiency.

PR precipitation was performed by adding a 4-fold volume of PR reagent to the protein supernatant, mixing thoroughly, and incubating at −20 °C for 30 min (or overnight). Precipitated proteins were collected by centrifugation at 19,000 × *g* for 10 min at 4 °C, and the pellets were washed twice with 2.5 volumes of ice-cold 95% ethanol. Protein pellets obtained from any method were resuspended in SDC buffer and subjected to ISD.

### Assessment of DNA and RNA Quality and Integrity

The quality and integrity of extracted DNA and RNA were assessed by agarose gel electrophoresis and capillary electrophoresis using an Agilent 5400 Bioanalyzer (Agilent Technologies) to determine the RNA integrity number (RIN). A 1% agarose gel was prepared in 1 × TAE buffer, stained with a nucleic acid dye (Biomed), and allowed to solidify at room temperature. DNA or RNA samples (5 μl) mixed with 6 × loading buffer (TIANGEN) were loaded alongside a DNA marker (TIANGEN). Electrophoresis was carried out in 1× TAE buffer at 210 V for DNA or 130 V for RNA for 15 min. Nucleic acid bands were visualized and recorded using a Tanon MINI Space Multifunctional Gel Imaging System

### Real-Time Reverse Transcription Polymerase Chain Reaction

To evaluate the quality of RNA extracted by π-SeqOmics from varying numbers of starting cells, the expression level of the housekeeping gene actin beta was quantified using quantitative RT-PCR. Total RNA was reverse transcribed into complementary DNA using the 5× FastKing-RT SuperMix (TIANGEN). Subsequently, quantitative RT-PCR amplification was performed on a CFX Opus 96 Real-Time PCR System (Bio-Rad) using SYBR Green reagents (TOYOBO). The following primer sequences were used for Actin:

Human-actin-forward: 5′–ATCACCATTGGCAATGAGCG–3′,

Human-actin-reverse: 5′–TTGAAGGTAGTTTCGTGGAT–3′.

### In-Solution Digestion

The ISD protocol was described by Gan *et al* (29). Protein pellets obtained from the multiomics extractions were first resuspended in SDC buffer. Both these resuspended protein solutions and the direct lysates from HEK 293T cells and mouse tissues were then subjected to reduction with 10 mM tris(2-carboxyethyl)phosphine and alkylation with 40 mM chloroacetamide at 65 °C for 10 min. Digestion was carried out by adding trypsin at a 1:50 enzyme-to-protein ratio and incubating at 37 °C for 14 h. The resulting peptides were desalted using homemade C18 StageTips. After desalting, a fraction of the peptides was dried in a centrifugal concentrator for full proteome analysis, while the remaining peptides were reserved for subsequent phosphopeptide enrichment.

### Phosphopeptides Enrichment

Phosphopeptides were enriched using an iron(III)-nitrilotriacetic acid (Fe-NTA)–based method as previously described (30). Briefly, 10 μl of Fe-NTA gel was added to the peptide solution, and the mixture was incubated with shaking at room temperature for 30 min. After centrifugation at 3000×*g* for 5 min, the supernatant was removed, and the gel was transferred onto a C8 membrane–based tip column. The column was pre-equilibrated with methanol and Fe-NTA loading buffer (80% acetonitrile [ACN], 0.1% TFA), washed twice with loading buffer, and bound phosphopeptides were eluted with Fe-NTA elution buffer (60% acetonitrile, 3% NH_3_·H_2_O). Eluates were dried in a SpeedVac concentrator and stored at −80 °C until LC-MS/MS analysis.

### π-SeqOmics

π-SeqOmics integrates column-based DNA/RNA coextraction with ZASP-mediated protein recovery, enabling the coordinated extraction of DNA, RNA, and proteins from a single sample. By partitioning peptides after digestion, this workflow enables both full proteome and phosphoproteome analyses.

The volumes of lysis buffer were adjusted according to the amount of input cells (see Supplementary Table S1). For approximately 10 mg of tissue samples, 350 μl of lysis buffer was used. The tissues were then homogenized at 7.1 m/s for three cycles of 10 s on and 10 s off. After homogenization, the samples were centrifuged at 19,000 × *g* for 5 min at 4 °C. DNA and RNA were isolated using a column-based DNA/RNA coextraction method, selected based on comparative evaluation for optimal compatibility with downstream proteomic analysis.

Proteins were recovered from the remaining fraction by ZASP precipitation (99.9% methanol, 0.1% FA, 200 mM ZnCl_2_), washed once with methanol/0.1% FA, and resuspended in SDC buffer. Proteins were then reduced, alkylated, and digested with trypsin overnight at 37 °C. Peptides were desalted using homemade C18 StageTips, after which approximately 10% of the digest was dried for global proteome analysis, and the remaining approximately 90% was enriched for phosphopeptides using Fe-NTA gel as previously described. The enriched phosphopeptides were dried and stored at −80 °C until LC–MS/MS analysis.

Detailed standard operating procedures for the entire π-SeqOmics workflow described above are provided in Supplemental Information S1.

### LC-MS/MS Analysis

For global proteome analysis, 100 ng of peptides were subjected to LC–MS/MS on an UltiMate 3000 RSLCnano system (Thermo Fisher Scientific) coupled to a timsTOF Pro mass spectrometer (Bruker). Peptides were separated on a 100 μm × 200 mm C18 analytical column (1.9 μm particles) at a flow rate of 300 nl/min using a 60-min linear gradient from 6% to 40% buffer B (0.1% FA in ACN). Data were acquired in data-independent acquisition with parallel accumulation–serial fragmentation mode over an *m/z* range of 300 to 1500. Ion mobility values were sampled from 1/K_0_ = 1.39 to 0.75 Vs/cm^2^, with both accumulation and ramp times set to 100 ms. Collision energy was dynamically adjusted in inverse proportion to ion mobility (59–20 eV across 1/K_0_ = 1.6–0.6 Vs/cm^2^).

For phosphoproteome analysis, 500 ng of enriched phosphopeptides (200 ng for sensitivity tests) were analyzed on an UltiMate 3000 RSLCnano system (Thermo Fisher Scientific) coupled to an Orbitrap Eclipse mass spectrometer (Thermo Fisher Scientific). Peptides were separated on a 100 μm × 300 mm C18 column (1.9 μm particles) at a flow rate of 300 nl/min using a linear gradient of 3% to 40% buffer B over 120 min, or 60 min for sensitivity tests. The Orbitrap was operated in data-dependent acquisition (DDA) mode with a 2-s duty cycle. Full MS scans were acquired at a resolution of 60,000 across an *m/z* range of 350 to 1500 with a maximum injection time of 45 ms, and MS/MS scans were acquired at a resolution of 30,000 using a 1.6 Da isolation window and a maximum injection time of 54 ms. Higher-energy collisional dissociation was applied with normalized collision energy set to 30%.

### Data Processing

For the database search, spectra were searched against the reviewed human (20,375 sequences, December 2021 release) or mouse (17,230 sequences, May 2025 release) FASTA reference proteomes obtained from UniProt. Databases were supplemented with common contaminants and reversed sequences for false discovery rate (FDR) estimation, and peptide and protein identifications were filtered at 1% FDR.

Raw data-independent acquisition with parallel accumulation–serial fragmentation data from full proteome analysis were processed in Spectronaut (v18.2, Biognosys) using default BGS factory settings, including dynamic retention time alignment and interference correction, with precursor and fragment mass tolerances set to 20 ppm. Label-free quantification (LFQ) was performed using the directDIA algorithm.

Raw DDA data from phosphoproteome analysis were searched with MSFragger implemented in FragPipe (v20.0) using the LFQ-phospho workflow. Searches were carried out with a precursor mass tolerance of 20 ppm, a fragment mass tolerance of 20 ppm, and a minimum peptide length of seven amino acids. Trypsin/P was specified as the protease, with up to two missed cleavages allowed. Carbamidomethylation of cysteine was set as a fixed modification, whereas oxidation of methionine, protein N-terminal acetylation, and phosphorylation of serine, threonine, and tyrosine (STY) were specified as variable modifications. LFQ was applied within the FragPipe workflow.

### Data Analysis

Data analysis and visualization were performed using R software (v4.4.1) (https://www.r-project.org), Microsoft Excel (v2024) (https://www.microsoft.com/en-us/microsoft-365/excel), and GraphPad Prism (v10.5) (https://www.graphpad.com/features). Only peptides and proteins with nonzero intensities were included, and phosphoproteomic data were filtered from FragPipe-generated STY.tsv files with site localization probability ≥ 0.75. Identification counts were compared using the Mann–Whitney *U* test for two groups and the Kruskal–Wallis test for multiple groups, with *p* values adjusted by the Benjamini–Hochberg method (FDR <0.05). Gene ontology (GO) enrichment analysis was carried out in DAVID Bioinformatics Resources (v6.8) using cellular component categories.

### Experimental Design and Statistical Rationale

All experiments were performed in triplicate (n = 3), with replicates prepared in parallel. For cell-based experiments, technical replicates were derived from the same biological source. For tissue-based applications, biological replicates were obtained from three independent dissections of the same tissue type. Equivalent numbers of HEK 293T cells or comparable tissue inputs were used across comparisons to ensure fair evaluation of workflow performance. The sample injection order was randomized to minimize instrument-related bias. Data quality and reproducibility were evaluated using SD, CV, and Pearson correlation coefficient. For statistical analyses, pairwise comparisons were assessed with the Mann–Whitney *U* test and multiple-group comparisons with the Kruskal–Wallis test; *p* values were adjusted for multiple testing using the Benjamini–Hochberg method, and results with FDR < 0.05 were considered significant. For phosphoproteomic analyses, only phosphosites with a localization probability > 0.75 were retained as confidently localized sites for downstream analysis.

## Results

### Benchmarking of Nucleic Acid Coextraction Methods for Proteome Compatibility

To establish a practical strategy for integrated multiomics workflows, we systematically compared three widely used nucleic acid extraction methods—TRIzol Reagent, a DR kit, and a DRP kit—to assess their compatibility with downstream proteomic analysis. Each method was processed according to its manufacturer’s instructions, using acetone or the kit-recommended PR reagent for protein precipitation, and the resulting proteomic data were compared with those obtained from the classical SDC-based ISD workflow. This evaluation focused on proteome depth, reproducibility, and quantitative consistency across methods.

**Fig. 1:**
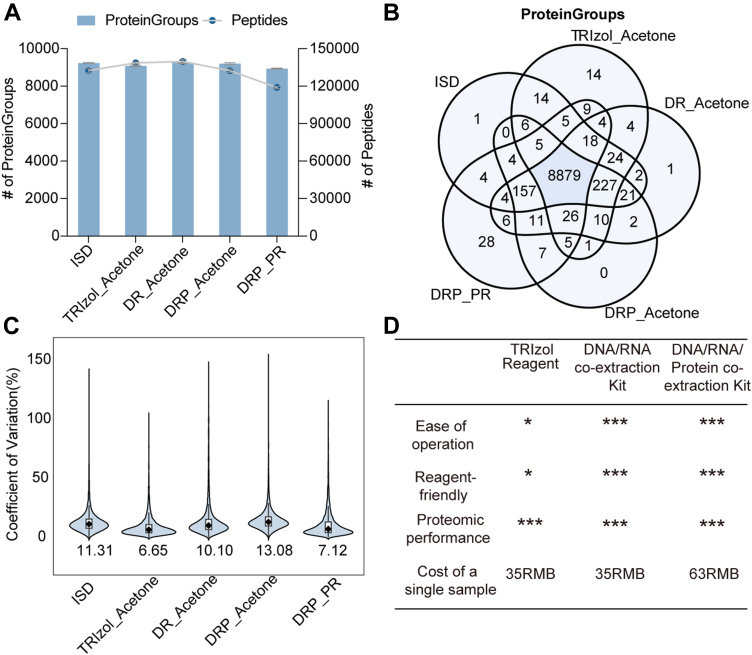
**Benchmarking of nucleic acid coextraction methods for proteome compatibility.***A*, numbers of identified protein groups and peptides obtained using the in-solution digestion (ISD) workflow and three nucleic acid extraction methods—TRIzol Reagent, DNA/RNA coextraction Kit (DR), and DNA/RNA/Protein coextraction Kit (DRP)—combined with acetone or the kit-recommended PR reagent for protein precipitation. *B*, Venn diagram illustrating overlap of identified protein groups among ISD, TRIzol–acetone, DR–acetone, DRP–acetone, and DRP–PR workflows. *C*, Violin plots showing the distribution of CVs for protein quantification across workflows. *D*, multidimensional comparison of extraction methods based on ease of operation, reagent compatibility, identification yield, and cost per sample.

Based on this design, peptides were analyzed by data-independent acquisition mass spectrometry on a timsTOF Pro system with a 60-min liquid chromatography (LC) gradient. All three extraction methods achieved deep proteome coverage, identifying more than 9000 proteins and 120,000 peptides—comparable to the ISD workflow. Among them, the DR kit showed the best performance, identifying an average of 9260 proteins and 139,794 peptides (Fig. 1*A*). The proprietary PR reagent used in the DRP kit yielded comparable results to acetone. Proteomes obtained across all conditions exhibited a 93% overlap (Fig. 1*B*), indicating that all workflows captured highly similar protein populations. Quantitative correlations with the ISD workflow were strong (r = 0.84–0.97; Supplementary Fig. S1), demonstrating consistent relative quantification across methods. Low CVs further confirmed the high reproducibility of all evaluated workflows (Fig. 1*C*), supporting their reliability for large-scale applications. Together, these results demonstrate that all three nucleic acid extraction methods are fully compatible with downstream proteomic analysis, achieving comparable depth and reproducibility to the classical ISD workflow.

To further evaluate the performance of the coextraction workflows under more demanding conditions, we compared the DR and DRP kits using mouse heart tissue, which represents a complex and difficult-to-lyse sample type. Consistent with the cell-based results, both workflows achieved proteomic depth comparable to the ISD control, with the DR kit identifying slightly more proteins (5485 *versus* 5431) and peptides (67,602 *versus* 65,639). Their proteomes showed 99% overlap and strong correlations with ISD. Regarding reproducibility, the DRP kit showed higher CVs than the ISD (Supplemental Fig. S2). Overall, when considering ease of operation, reagent compatibility, proteomic performance, and cost (Fig. 1*D*), the DR kit emerged as the most balanced and practical option for multiomics sample preparation. We therefore selected the DR workflow as the standard for subsequent multiomics analyses.

### Benchmarking ZASP and Acetone Precipitation for Protein Recover

Building on the optimized nucleic acid coextraction workflows established above, we next focused on improving the protein recovery step. Acetone precipitation is widely used for this purpose, but requires long incubation and centrifugation steps. Given our prior development of the zinc-assisted sample preparation (ZASP) method—a rapid ZnCl_2_/methanol-based precipitation approach—we evaluated whether ZASP could serve as a faster and equally effective alternative to acetone for recovering proteins from the effluents of nucleic acid extraction. Peptides generated from both precipitation methods were analyzed by data-independent acquisition mass spectrometry following the same analytical workflow used in the preceding section.

**Fig. 2:**
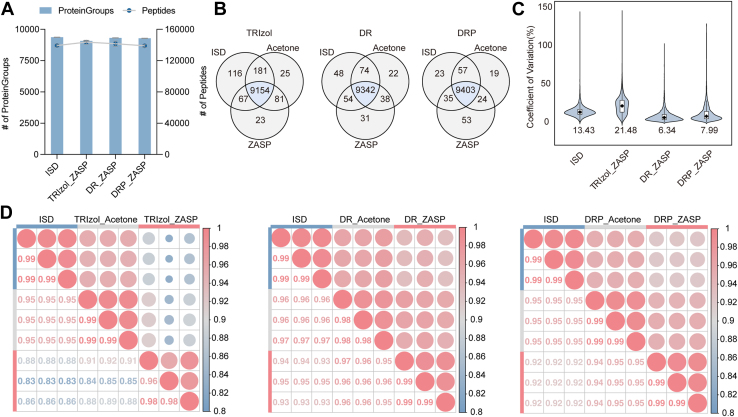
**Comparison of ZASP and acetone precipitation methods for protein recovery from nucleic acid extraction effluents.***A*, number of identified protein groups and peptides obtained using the ISD workflow and each coextraction workflow combined with acetone or ZASP precipitation. *B*, Venn diagrams showing the overlap of identified proteomes across ISD, acetone, and ZASP workflows for TRIzol, DR, and DRP extractions. *C*, Violin plots illustrating the distribution of CVs for quantitative reproducibility. (*D*) Pairwise Pearson correlation matrices of protein intensities comparing ISD, acetone, and ZASP workflows for each extraction method. DR, DNA/RNA coextraction Kit; DRP, DNA/RNA/Protein coextraction Kit; ISD, in-solution digestion.

Quantitative analysis showed that ZASP precipitation achieved proteome coverage comparable to acetone precipitation across all three nucleic acid extraction workflows and the ISD workflow (Fig. 2*A*). Each condition identified more than 9000 proteins and approximately 130,000 peptides. The identified proteomes from acetone, ZASP, and ISD showed extensive overlap, with more than 9000 proteins shared across workflows and the lowest overlap still reaching 97% (Fig. 2*B*). In terms of reproducibility, protein quantification showed low CVs across all workflows, with median values of 13.4% for ISD, 21.5% for TRIzol–ZASP, 6.3% for DR–ZASP, and 8.0% for DRP–ZASP (Fig. 2*C*). Correlation analysis further confirmed strong consistency among the workflows (Fig. 2*D*). The acetone- and ZASP-based DR and DRP workflows were highly correlated with ISD (r > 0.95), whereas the TRIzol–ZASP workflow showed lower correlation (r ≈ 0.84) and higher variability. Both observations likely result from reduced precipitation efficiency in TRIzol effluents under acidic conditions (pH 5.0), consistent with ZASP's known preference for mildly alkaline environments (pH 8.5). Finally, GO cellular component analysis revealed nearly identical subcellular distribution patterns of proteins identified from acetone- and ZASP-precipitated samples across all extraction workflows (Supplementary Fig. S3). Together, these results demonstrate that the ZASP method provides a rapid and efficient alternative to acetone precipitation, achieving equivalent proteome depth, reproducibility, and quantitative consistency while offering advantages in processing time and scalability.

### Development of an Integrated Multiomics Workflow—π-SeqOmics

**Fig. 3:**
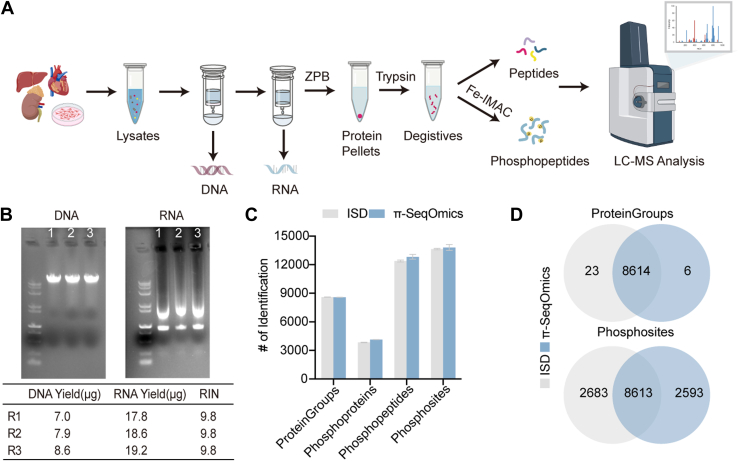
**Development and performance evaluation of the integrated multiomics workflow—π-SeqOmics.***A*, schematic overview of the π-SeqOmics workflow. DNA and RNA are isolated using the DNA/RNA coextraction Kit, while proteins remaining in the effluent are recovered through ZASP-based precipitation, digested with trypsin, and divided into total peptides and phosphopeptides via Fe-IMAC enrichment for LC–MS/MS analysis. *B*, evaluation of nucleic acid extraction performance. The upper panels show agarose-gel electrophoresis, while the *lower table* summarizes the correspondi—ng DNA yield, RNA yield, and RNA integrity number (RIN) obtained in three technical replicates. *C*, comparison of proteome and phosphoproteome depth between π-SeqOmics and the classical ISD workflow. *D*, Venn diagrams showing the overlap of protein groups and phosphosites identified by π-SeqOmics and ISD. IMAC, immobilized metal affinity chromatography; ISD, in-solution digestion; LC, liquid chromatography.

Integrating the optimized DNA/RNA coextraction and ZASP-based protein recovery strategies, we developed π-SeqOmics, an integrated workflow that enables the sequential isolation of DNA, RNA, total peptides, and phosphopeptides from a single sample. In this workflow, DNA and RNA are separated using the DR, followed by ZASP precipitation of proteins from the remaining effluent. The recovered proteins are digested in solution, with 10% of peptides analyzed for global proteomics and the remaining 90% enriched for phosphopeptides using an Fe-NTA gel (Fig. 3*A*). A detailed, complete experimental workflow for π-SeqOmics is provided in Supplementary Fig. S4. To validate its performance, π-SeqOmics was compared with the classical ISD workflow using triplicate HEK293T samples.

The performance of π-SeqOmics in nucleic acid extraction was first evaluated to confirm its suitability for integrated multiomics analysis. From 5 × 10^6^ HEK293T cells, the workflow yielded an average of 7.8 μg DNA and 18.5 μg RNA. Agarose gel electrophoresis and RIN analysis (average RIN = 9.8) verified that the extracted nucleic acids met the quality requirements for whole-genome and transcriptome (RNA-seq) sequencing, respectively (Fig. 3*B*). After confirming high-quality nucleic acid recovery, we next assessed proteomic performance. π-SeqOmics identified 8584 proteins and 122,545 peptides—closely matching ISD results (8603 proteins, 124,444 peptides). The two workflows exhibited extensive overlap, sharing 99.7% of proteins and 98.2% of peptides (Fig. 3, *C*, *D* and Supplementary Fig. S5*A*), indicating that nucleic acid extraction did not compromise protein recovery or the depth of protein identification. π-SeqOmics demonstrated strong quantitative concordance with ISD (r > 0.94) and highly consistent replicate performance across runs (r ≥ 0.97; Supplementary Fig. S5*B*). GO cellular component analysis further confirmed highly similar subcellular proteome distributions between π-SeqOmics and ISD (Supplementary Fig. S5*C*).

For phosphoproteomic analysis, only phosphosites with localization probabilities ≥ 0.75 were considered for quantification and downstream analysis. π-SeqOmics identified 13,806 phosphosites, 12,826 phosphopeptides, and 4141 phosphoproteins—closely matching ISD results (13,640 phosphosites, 12,385 phosphopeptides, and 3841 phosphoproteins). The two workflows showed substantial overlap, sharing 71.0% of phosphosites, 72.5% of phosphopeptides, and 82.9% of phosphoproteins (Fig. 3, *C*, *D* and Supplementary Fig. S5*E*). Pearson correlations between π-SeqOmics and ISD ranged from 0.82 to 0.88, while replicate correlations within each workflow exceeded 0.97 (Supplementary Fig. S5*F*). Phosphosite intensity distributions were highly consistent (median log_10_ intensity was 8.27 compared to 8.17; Supplementary Fig. S5*D*). GO analysis revealed that π-SeqOmics detected slightly more phosphoproteins within the top 10 cellular compartments (Supplementary Fig. S5*G*), and the ratios of phosphorylated serine, threonine, and tyrosine residues were nearly identical between workflows (571:53:1 *versus* 574:50:1; Supplementary Fig. S5*H*).

Taken together, these results demonstrate that π-SeqOmics consistently yields high-quality proteome and phosphoproteome data. This robust and efficient workflow enables simultaneous analysis of the genome, transcriptome, proteome, and phosphoproteome from a single biological sample, providing a practical solution for integrated multiomics studies.

### Sensitivity of π-SeqOmics Across Different Sample Input Amounts

**Fig. 4:**
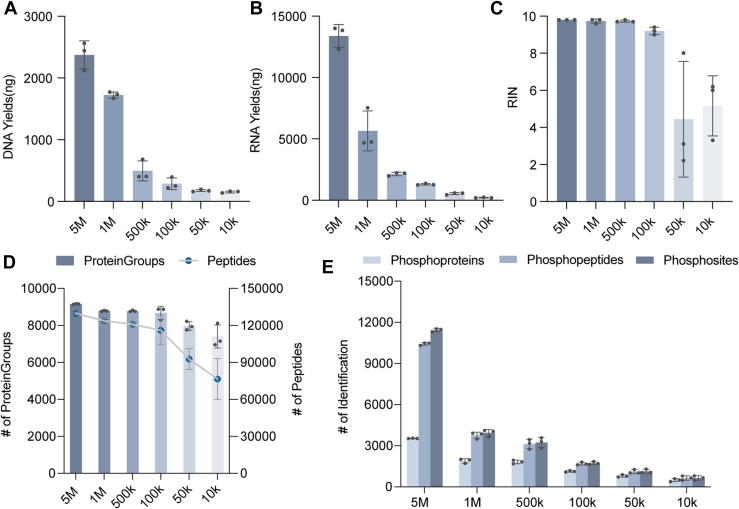
**Sensitivity of π-SeqOmics across different sample input amounts.***A*, DNA yields obtained from HEK293T cells at six input levels ranging from 5 × 10^6^ to 1 × 10^4^ cells. *B*, RNA yield measured from HEK293T cells at corresponding input levels. *C*, RIN values measured from HEK293T cells at corresponding input levels. *D*, the number of identified protein groups and peptides across different input amounts. *E*, the number of identified phosphoproteins, phosphopeptides, and phosphosites across different input amounts. All data represent mean ± SD from three biological replicates. RIN, RNA integrity number; HEK, human embryonic kidney.

To evaluate the sensitivity of π-SeqOmics across different sample amounts, HEK293T cells were processed at six input levels ranging from 5 × 10^6^ to 1 × 10^4^ cells, with three biological replicates per group. DNA and RNA yields decreased proportionally with reduced cell numbers (Fig. 4, *A* and *B*). From 5 × 10^6^ cells, π-SeqOmics recovered an average of 2.3 μg DNA and 13.5 μg RNA, while 1 × 10^4^ cells still yielded 111 ng DNA and 213 ng RNA. Agarose-gel electrophoresis showed that both DNA and RNA remained intact across the tested input range (Supplementary Fig. S6*A*). RNA quality was further assessed by RIN analysis, which indicated consistently high-quality RNA recovery, with RIN values > 9.0 for samples containing ≥ 1 × 10^5^ cells (Fig. 4*C*). Although RIN values exhibited a decline and increased variability at ultra-low inputs, qRT-PCR of the housekeeping gene actin beta produced cycle threshold values below 35 for all samples, indicating that the recovered RNA remains suitable for downstream amplification even at these limits (Supplementary Fig. S6*B*).

For the proteome (Fig. 4*D*), π-SeqOmics maintained high identification depth and quantitative consistency across a 1000-fold range of input amounts. Protein and peptide identifications remained largely stable between 1 × 10^5^ and 1 × 10^6^ cells—the range also recommended for reliable DNA/RNA coextraction in multiomics workflows. Within this interval, π-SeqOmics identified 9144 proteins and 130,589 peptides from 5 × 10^6^ cells, and comparable numbers were obtained at 1 × 10^6^ (8792 proteins; 121,879 peptides), 5 × 10^5^ (8682; 114,750), and 1 × 10^5^ cells (8761; 118,331). When the input dropped below 1 × 10^5^ cells, identification counts decreased sharply, with 7911 ± 268 proteins and 96,724 ± 9065 peptides detected at 5 × 10^4^ cells, and 7419 ± 577 proteins and 77,339 ± 15,685 peptides at 1 × 10^4^ cells. For the inputs from different cells, the within-group Pearson's correlation coefficient exceeded 0.92 (Supplementary Fig. S6*C*). The increased variability at these ultra-low inputs (Supplementary Fig. S6*D*) reflects an inherent limitation commonly observed in low-input proteomic analyses. Despite reduced identification depth, π-SeqOmics retained broad quantitative coverage even at minimal sample amounts. Across all input levels, rank–abundance curves showed comparable intensity distributions spanning more than 5 orders of magnitude (Supplementary Fig. S6*E*), highlighting the method’s ability to preserve a broad dynamic range across diverse sample inputs.

For the phosphoproteome (Fig. 4*E*), analyses were performed using a 60-min LC gradient in DDA mode. At an input of 5 × 10^6^ cells, π-SeqOmics identified 3533 phosphoproteins, 10,427 phosphopeptides, and 11,435 phosphosites. The number of identifications decreased with reduced input, showing a marked drop from 1 × 10^6^ to 1 × 10^5^ cells. Compared with the global proteome, phosphoproteome coverage was more sensitive to input reduction. All samples were processed using the same enrichment and LC–MS conditions, which were not specifically optimized for lower input levels. These results reflect the current, nonoptimized state of the workflow, with room for improvement in phosphopeptide enrichment and LC–MS sensitivity for low-input samples. Collectively, these results indicate that the current π-SeqOmics workflow performs efficiently and reproducibly for integrated multiomics analysis with ≥ 1 × 10^5^ cells, and can be further optimized for lower-input applications.

### Applicability of π-SeqOmics Across Diverse Mouse Tissues

**Fig. 5:**
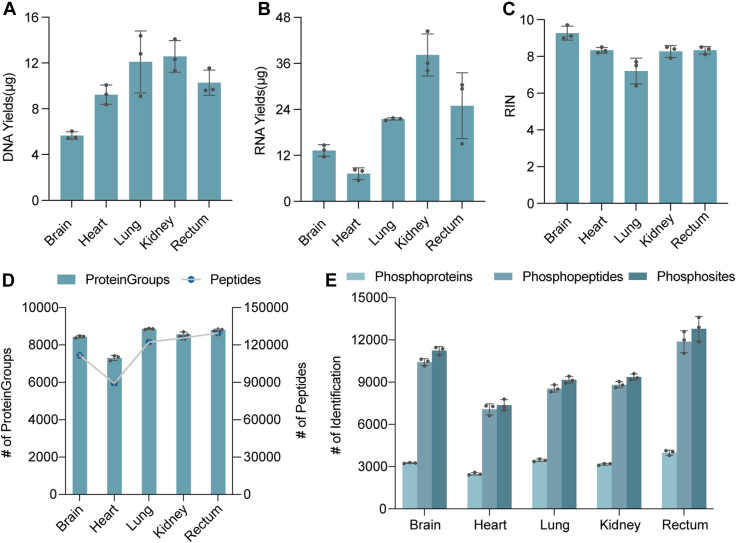
**Applicability of π-SeqOmics across diverse mouse tissues.***A*, DNA yields from five freshly frozen mouse tissues (brain, heart, lung, kidney, and rectum) processed using π-SeqOmics. *B*, RNA yields across different tissues. *C*, RIN values across the 5 mouse tissues. *D*, the number of identified protein groups and peptides across different tissues. *E*, the number of identified phosphoproteins, phosphopeptides, and phosphosites across the five tissues. All data are presented as mean ± SD from three biological replicates (approximately 10 mg of tissue per replicate). RIN, RNA integrity number.

To evaluate the applicability of π-SeqOmics across different biological matrices, we applied it to five freshly frozen mouse tissues—brain, heart, lung, kidney, and rectum—for the sequential isolation of DNA, RNA, and Proteins. Three biological replicates (∼10–12 mg each) were homogenized by grinding prior to extraction to ensure efficient tissue disruption. DNA and RNA yields varied by tissue, ranging from 6 μg DNA and 13 μg RNA in the brain to 13 μg DNA and 38 μg RNA in the kidney (Fig. 5, *A* and *B*). Agarose-gel electrophoresis showed that both DNA and RNA remained intact in all five tissues. RNA quality was further evaluated by RIN analysis, which yielded values ranging from 6.4 to 9.7 across tissues. Brain tissue showed the highest RNA integrity (average RIN = 9.3), whereas lung tissue exhibited slightly greater variation but still remained suitable for downstream applications (Fig. 5*C* and Supplementary Fig. S7).

At the proteomic level, π-SeqOmics achieved in-depth coverage across all tissues, identifying more than 8000 proteins and 110,000 peptides in most tissues, with slightly lower numbers in the heart (average 7307 proteins and 89,155 peptides; Fig. 5*D*). Replicate analyses showed high consistency, with Pearson correlation coefficients exceeding 0.95 and low coefficients of variation across all tissues (Supplementary Fig. S8, *A* and *B*), indicating robust and reproducible proteomic performance across diverse tissue types. For phosphoproteomic analysis, π-SeqOmics also achieved broad and consistent coverage across all tissues, with the rectum yielding the highest number of identifications (3189 phosphoproteins, 7256 phosphopeptides, and 9103 phosphosites), and the heart showing the lowest (1879 phosphoproteins, 4227 phosphopeptides, and 5043 phosphosites; Fig. 5*E*). Correlation analyses of phosphosite intensities demonstrated high reproducibility across replicates (r ≥ 0.85; Supplementary Fig. S8*C*), with moderate variation in phosphopeptide enrichment efficiency among tissues (Supplementary Fig. S8*D*). Together, these results show that π-SeqOmics supports the recovery of sequencing-quality DNA and RNA while enabling sensitive, reproducible, and in-depth proteomic and phosphoproteomic profiling across diverse mouse tissues.

## Discussion

Obtaining high-quality multiomics data from a single limited specimen remains a central challenge in systems biology and precision medicine (31, 32). Conventional aliquoting or parallel extraction strategies often introduce biological bias and hinder accurate data integration, particularly when working with scarce or heterogeneous clinical materials (33). Although several prior studies, including TRIzol- and kit-based protocols, have established the feasibility of sequentially recovering DNA, RNA, and proteins from the same specimen, their compatibility with deep proteomic and phosphoproteomic analyses has not been systematically evaluated. In addition, most existing approaches rely on multi-step extraction procedures involving phenol-based reagents, which make them difficult to standardize or automate, thus unsuitable for large-scale implementation (20, 21, 25, 34).

To address these limitations, we systematically benchmarked representative nucleic acid extraction workflows for their compatibility with downstream proteomic analyses. All tested methods achieved proteome coverage comparable to conventional ISD, confirming that concurrent nucleic acid extraction does not compromise proteomic depth or reproducibility. Among them, the DR exhibited the most balanced performance in terms of yield, reproducibility, ease of use, and cost-effectiveness, establishing a practical foundation for integrated multi-omics workflows. In parallel, replacing conventional acetone precipitation with the ZASP method significantly simplified protein recovery, reduced handling complexity, and eliminated phenol-based reagents, while maintaining equivalent proteomic performance.

Building upon these optimizations, we developed π-SeqOmics, a unified and phenol-free workflow that enables sequential isolation of DNA, RNA, and proteins from a single sample using accessible reagents and uniform procedures. Benchmarking against the classical ISD workflow demonstrated comparable identification depth and quantitative reproducibility across all omics layers. π-SeqOmics maintained consistent multiomics performance down to approximately 1 × 10^5^ cells, which also represents the typical lower limit for reliable DNA/RNA coextraction. Below this range, proteomic and phosphoproteomic coverage declined more markedly, reflecting the increased sensitivity of peptide-level analyses to reduced input amounts. Extending the validation to multiple mouse tissues, π-SeqOmics consistently yielded sequencing-grade nucleic acids and reproducible proteomic and phosphoproteomic profiles, identifying over 8000 proteins in most tissues with replicate correlations exceeding 0.95.

In summary, π-SeqOmics provides a simple, reproducible, and cost-effective solution for comprehensive multiomics profiling from a single specimen. By systematically benchmarking nucleic acid extraction and protein recovery strategies, π-SeqOmics establishes a standardized workflow for integrated analyses from limited-input samples. Its simplicity, robustness, and reagent accessibility make it a practical foundation for cross-laboratory multiomics sample preparation—supporting reproducible molecular data generation for systems biology, biomarker discovery, and translational research.

## Data Availability

The proteomic data have been deposited in the ProteomeXchange Consortium (https://proteomecentral.proteomexchange.org) via the iProX partner repository (35, 36) with the dataset identifier PXD069575. Reviewers can access the data via https://www.iprox.cn/page/SSV024.html;url=1760686718171CYAi with the password “Z3bh.”

## Supplemental data

This article contains supplemental data.

## Conflict of Interest

The authors declare no competing interests.
