# Supplementary material for: π-SeqOmics: A Sequential Workflow for Genomic, Transcriptomic, Proteomic, and Phosphoproteomic Profiling From Biopsy-Scale Samples
Source: Mol Cell Proteomics. 2026 May 28;25(7):101596. doi: 10.1016/j.mcpro.2026.101596 (PMC13312471; doi:10.1016/j.mcpro.2026.101596)
Supplement: Supplemental Figures [file mmc1.docx]

**Supplemental Figures**

**π-SeqOmics: A Sequential Workflow for Genomic, Transcriptomic, Proteomic and Phosphoproteomic Profiling from Biopsy-Scale Samples**

Shuyi Feng^2,3^, Xuehui Deng^3,4^, Ying Xu^3^, Baoyi Qin^3^, Chuanxi Huang^3^, Qingjing Chen^3,4^, Fuchu He^1,2,3 *^, and Dongxue Wang^1,3 *^

1 State Key Laboratory of Medical Proteomics, Beijing Proteome Research Center, National Center for Protein Sciences (Beijing), Research Unit of Proteomics-driven Cancer Precision Medicine (Chinese Academy of Medical Sciences), Beijing Institute of Lifeomics, Beijing 102206, China

2 Department of Chemistry, School of Science, Southern University of Science and Technology, Shenzhen 518055, China

3 International Academy of Phronesis Medicine, Guangzhou 510005, Guangdong, China

4 Nanfang Hospital, Southern Medical University, Guangzhou 510515, China

*Correspondence to:

Fuchu He: hefc@bmi.ac.cn

Dongxue Wang: [wang_dongxue@126.com](mailto:wang_dongxue@126.com)

**Figure S1-2:** Benchmarking of nucleic acid co-extraction methods for proteome compatibility

**Figure S3:** Comparison of ZASP and acetone precipitation methods for protein recovery from nucleic acid extraction effluents.

**Figure S4:** Comprehensive workflow of π-SeqOmics illustrating the detailed experimental procedures.

**Figure S5:** Performance evaluation and benchmarking of π-SeqOmics.

**Figure S6:** Sensitivity of π-SeqOmics across different sample input amounts

**Figure S7-8:** Applicability of π-SeqOmics across Diverse Mouse Tissues


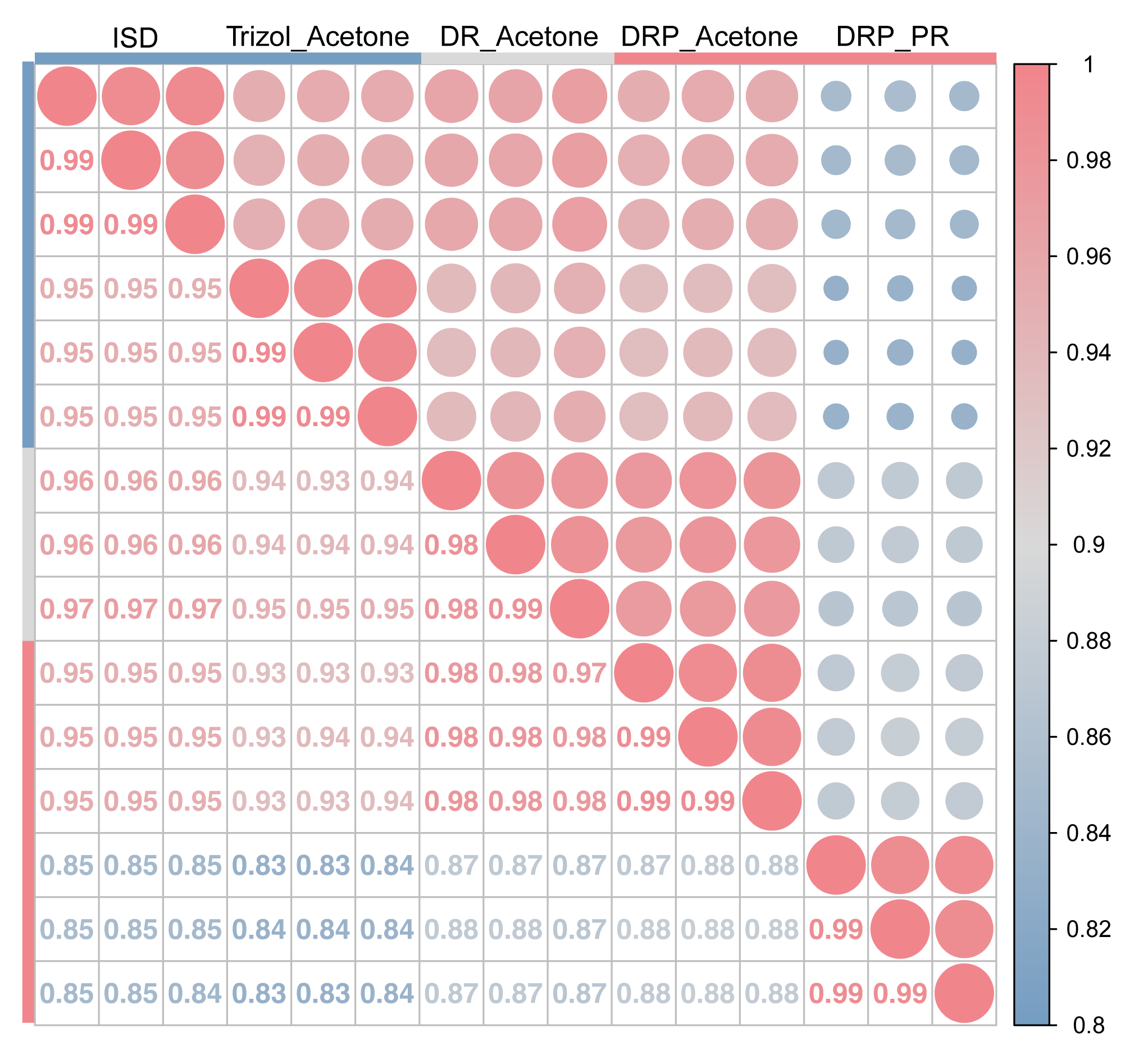


**Figure S1.** Benchmarking of nucleic acid co-extraction methods for proteome compatibility. Pearson correlation coefficients for protein identifications among the three nucleic acid separation methods and ISD.


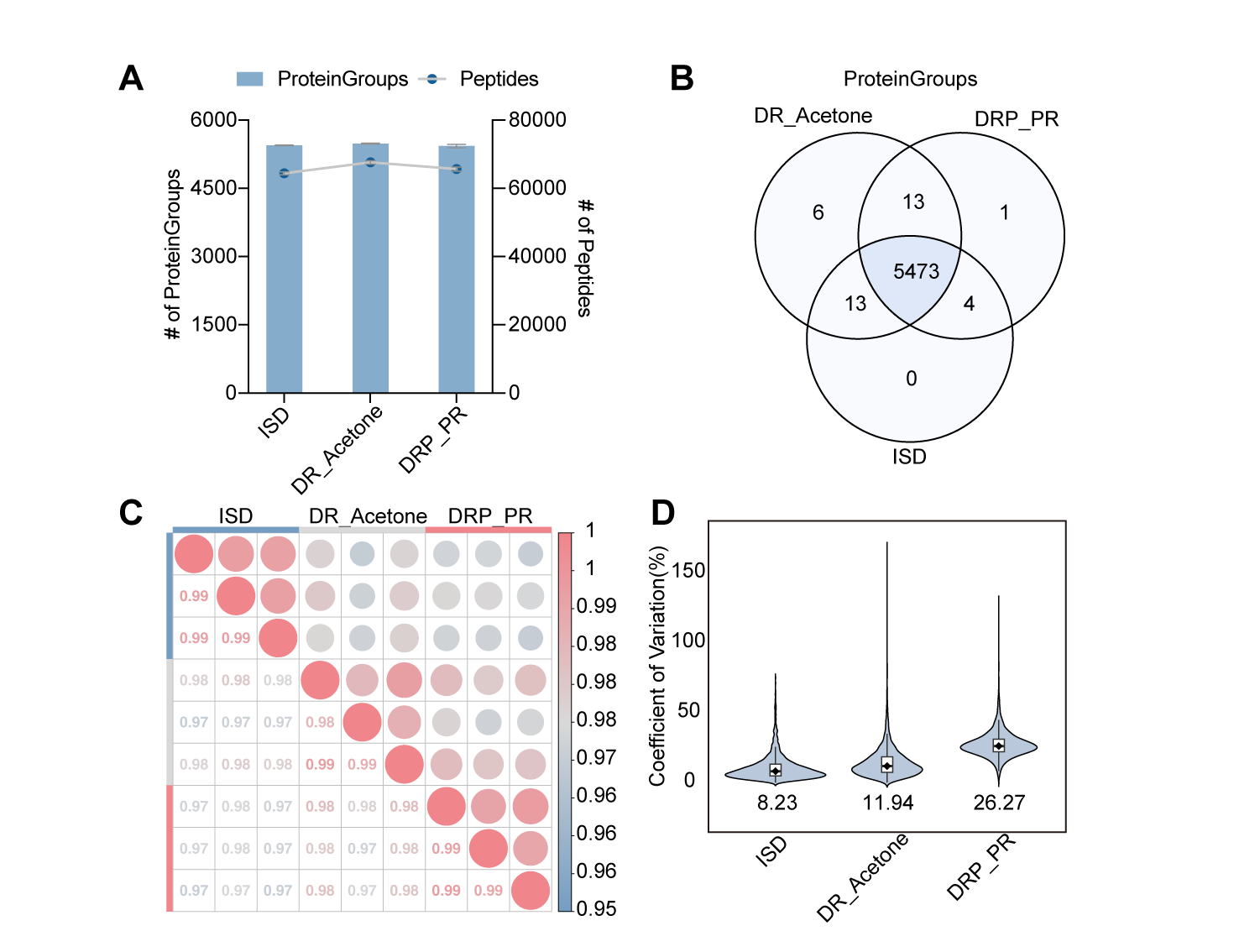


**Figure S2.** Benchmarking of nucleic acid co-extraction methods for proteome compatibility. (A) Comparison of protein and peptide identification numbers among DR, DRP, and ISD. (B) Venn diagram showing the overlap of identified proteins among the two nucleic acid separation methods and ISD. (C) Pearson correlation coefficients for protein identifications among the two nucleic acid separation methods and ISD. (D) Coefficients of Variation(CVs) for proteins among the three methods.


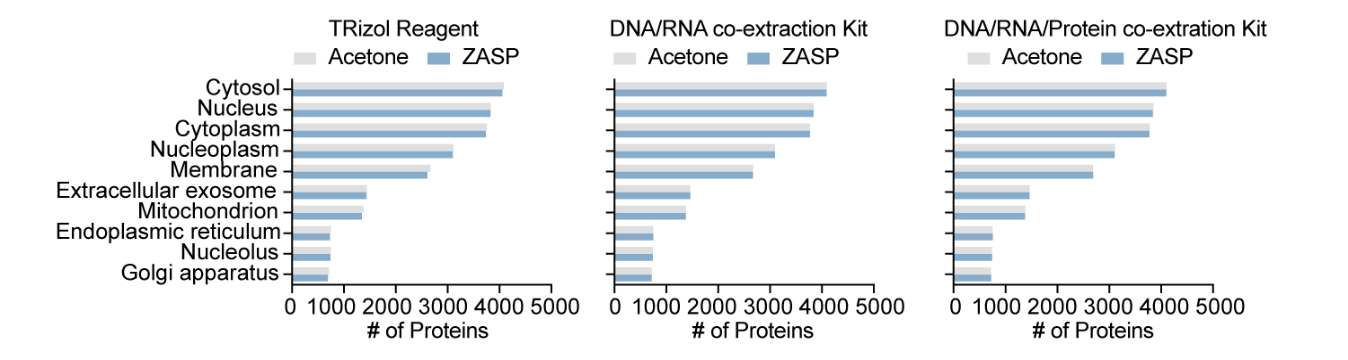


**Figure S3.** Comparison of ZASP and acetone precipitation methods for protein recovery from nucleic acid extraction effluents. Gene Ontology (GO) Cellular Component annotation of identified proteins, comparing acetone and ZASP precipitation within each of the three nucleic acid separation methods.


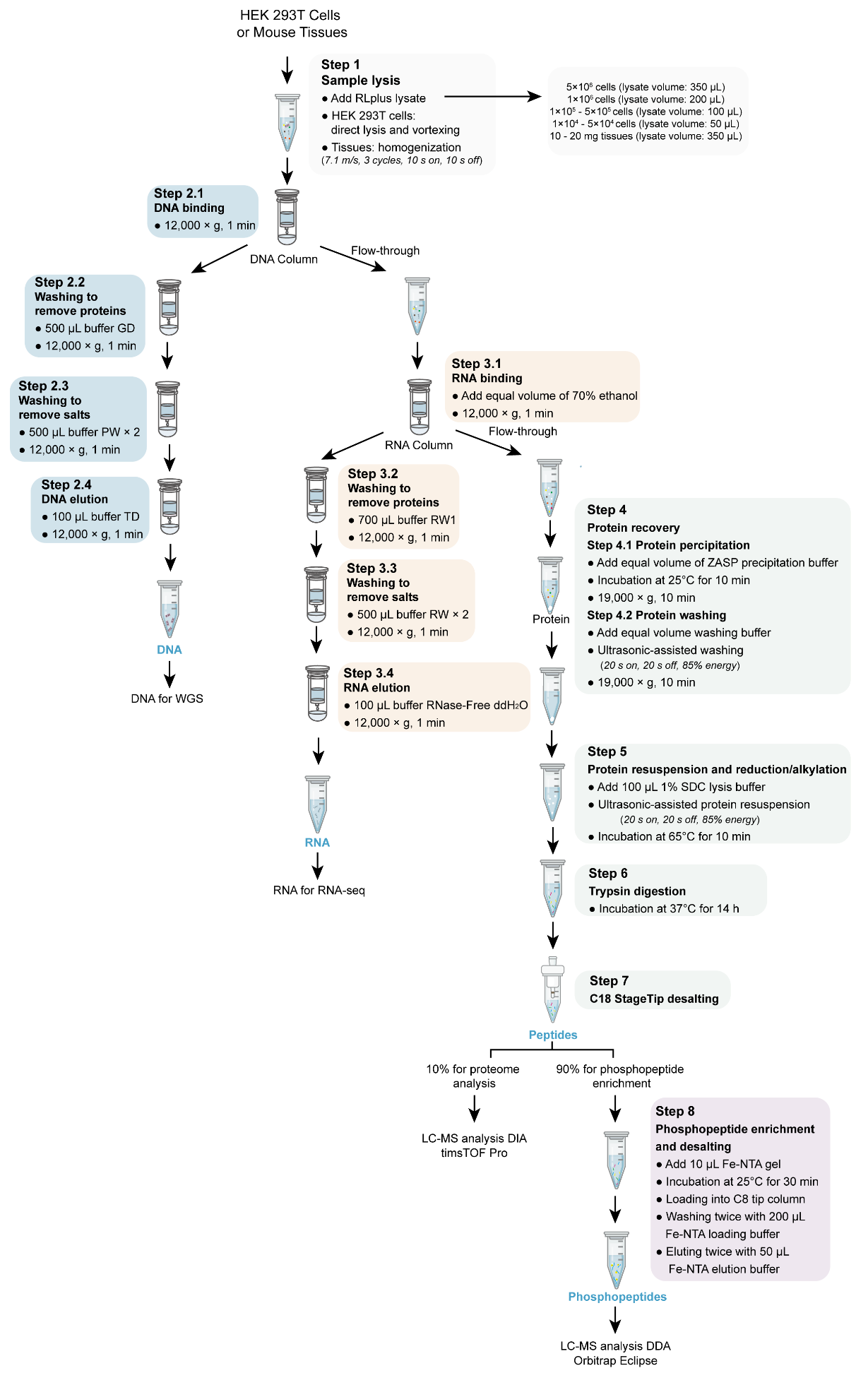


**Figure S4.** Comprehensive workflow of π-SeqOmics illustrating the detailed experimental procedures.


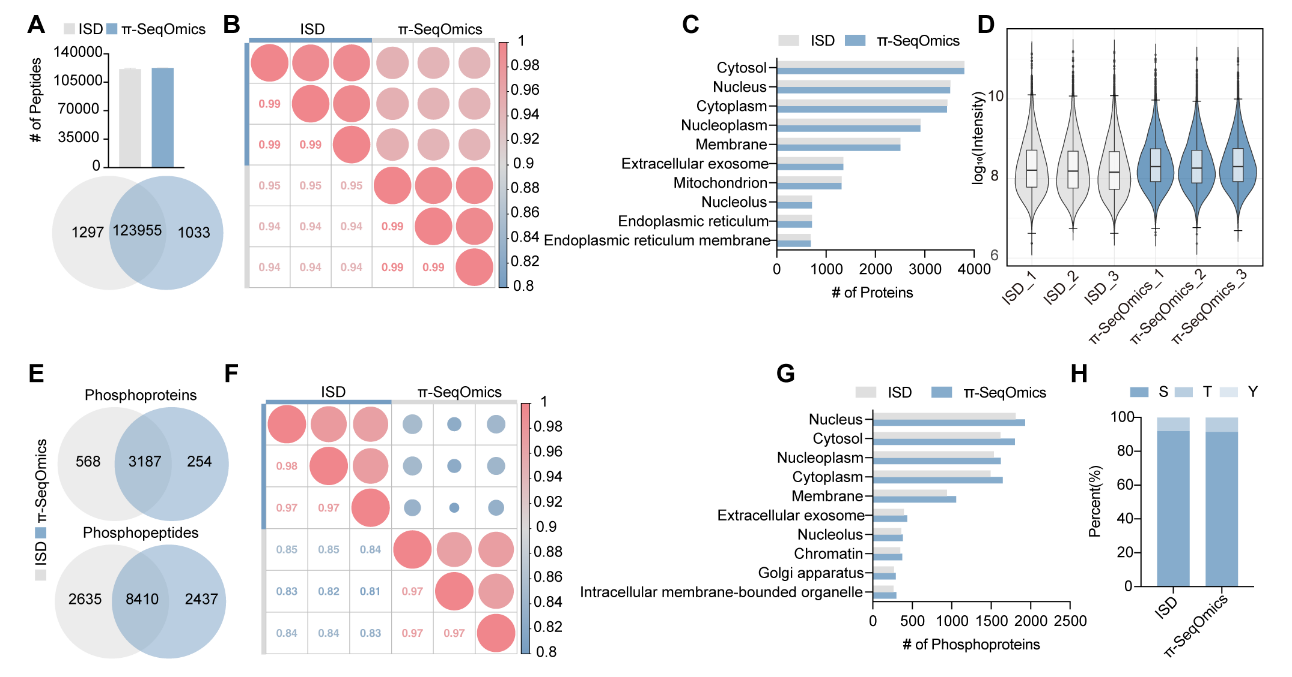


**Figure S5.** Performance evaluation and benchmarking of π-SeqOmics. (A) Peptide identification numbers and overlap analysis of the ISD and the π-SeqOmics. (B) Pearson‘s correlation coefficients for proteins identified by ISD and the π-SeqOmics. (C) GO Cellular Component annotation for proteins identified by ISD and the π-SeqOmics. (D) Violin plots show the log_10_ mean intensities of identified phosphosites with loc. prob. >0.75 between ISD and the π-SeqOmics. (E) Venn diagram showing the overlap of identified phosphoproteins and phosphopeptides between ISD and π-SeqOmics. (F) Pearson‘s correlation coefficients for Phosphosites identified by ISD and the π-SeqOmics. (G) GO Cellular Component annotation for phosphoproteins identified by ISD and the π-SeqOmics. (H) The number of identified peptides containing STY amino acids between ISD and π-SeqOmics.


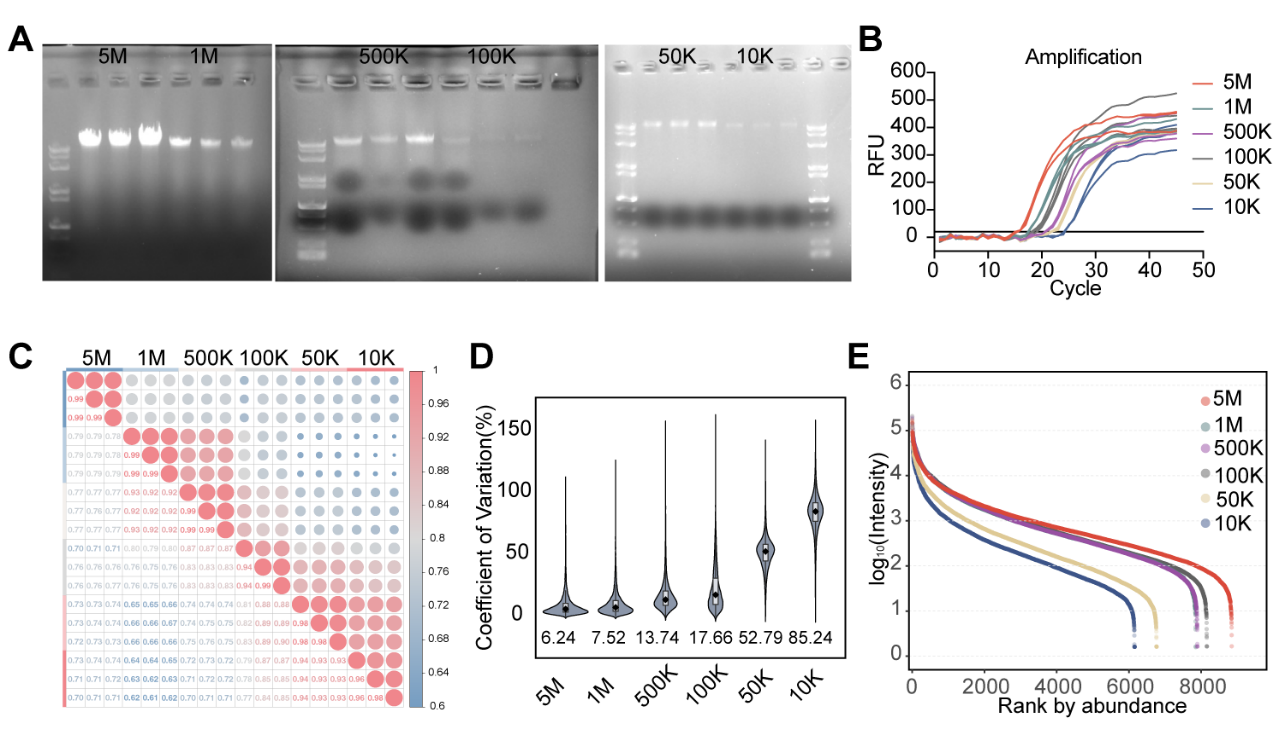


**Figure S6.** Sensitivity of π-SeqOmics across different sample input amounts**.** (A) Agarose gel electrophoresis results for DNA from different HEK 293T cell input amounts. (B) Quality assessment of RNA extracted from various HEK 293T cell input amounts via qRT-PCR using ACTB as a housekeeping gene. (C) Pearson correlation coefficients for protein identifications from various HEK 293T cell input amounts. (D) Violin plots showing the distribution of CVs for protein quantification across various HEK 293T cell input amounts. (E) Ranked plots illustrating protein abundance across various HEK 293T cell input amounts.


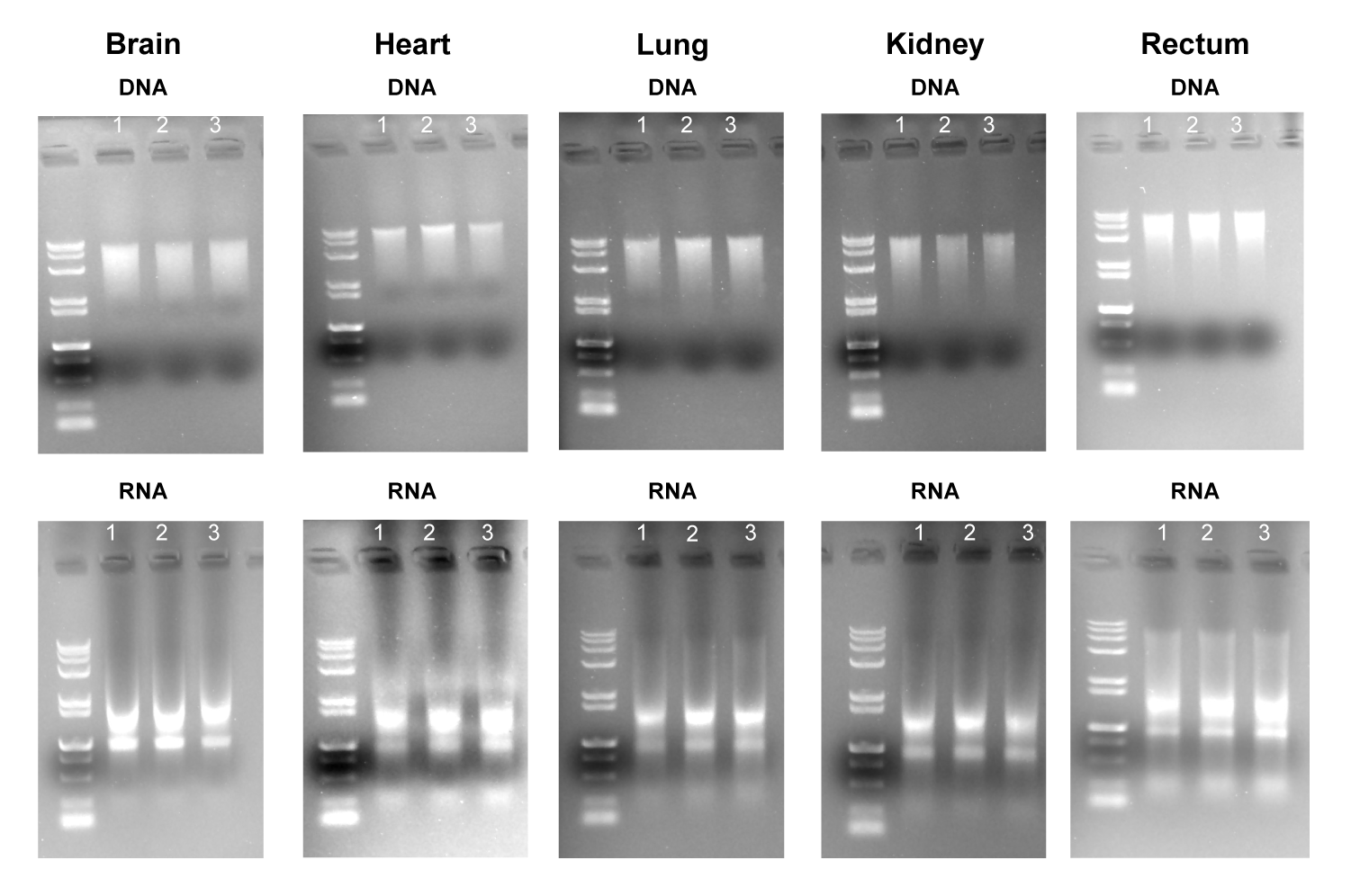


**Figure S7.** Applicability of π-SeqOmics across Diverse Mouse Tissues. Agarose gel electrophoresis results for DNA and RNA from five different fresh-frozen mouse tissues.


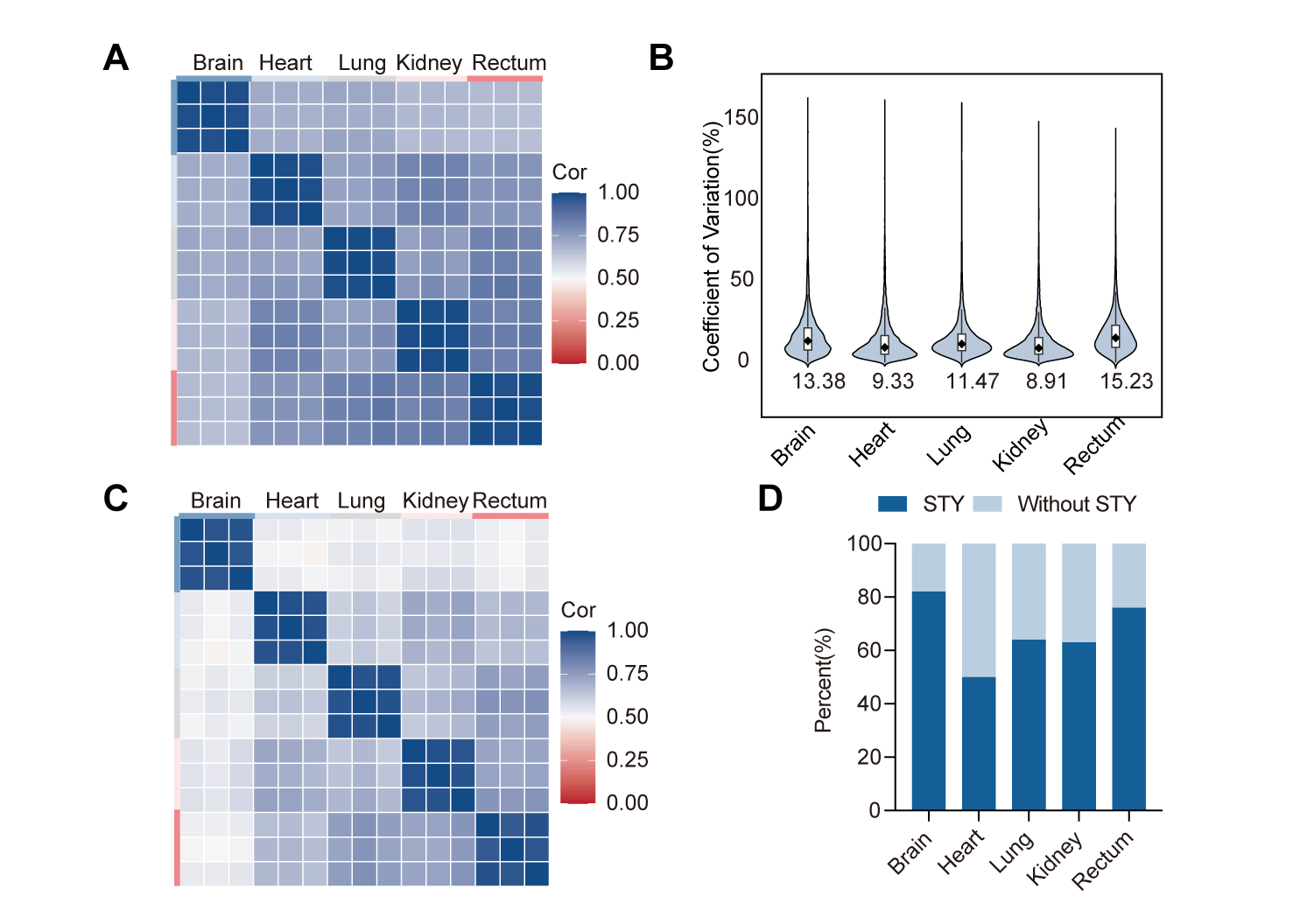


**Figure S8.** Applicability of π-SeqOmics across Diverse Mouse Tissues. (A) Pearson correlation coefficients for protein identifications from five different fresh-frozen mouse tissues. (B) Coefficients of variation for proteins from five different fresh-frozen mouse tissues. (C)Pearson correlation coefficients for phosphosites identifications from five different fresh-frozen mouse tissues. (D) Phosphopeptides enrichment efficiency from five different fresh-frozen mouse tissues.
