# Supplementary material for: π-SeqOmics: A Sequential Workflow for Genomic, Transcriptomic, Proteomic, and Phosphoproteomic Profiling From Biopsy-Scale Samples
Source: Mol Cell Proteomics. 2026 May 28;25(7):101596. doi: 10.1016/j.mcpro.2026.101596 (PMC13312471; doi:10.1016/j.mcpro.2026.101596)
Supplement: Supplementary Information S1 [file mmc2.docx]

**Supplementary Information S1**

**π-SeqOmics: A Sequential Workflow for Genomic, Transcriptomic, Proteomic and Phosphoproteomic Profiling from Biopsy-Scale Samples**

Shuyi Feng^2,3^, Xuehui Deng^3,4^, Ying Xu^3^, Baoyi Qin^3^, Chuanxi Huang^3^, Qingjing Chen^3,4^, Fuchu He^1,2,3 *^, and Dongxue Wang^1,3 *^

1 State Key Laboratory of Medical Proteomics, Beijing Proteome Research Center, National Center for Protein Sciences (Beijing), Research Unit of Proteomics-driven Cancer Precision Medicine (Chinese Academy of Medical Sciences), Beijing Institute of Lifeomics, Beijing 102206, China

2 Department of Chemistry, School of Science, Southern University of Science and Technology, Shenzhen 518055, China

3 International Academy of Phronesis Medicine, Guangzhou 510005, Guangdong, China

4 Nanfang Hospital, Southern Medical University, Guangzhou 510515, China

*Correspondence to:

Fuchu He: hefc@bmi.ac.cn

Dongxue Wang: [wang_dongxue@126.com](mailto:wang_dongxue@126.com)

Supplemental Table

**Table S1:** Detailed parameters of the optimized experimental system for nucleic acid extraction and protein precipitation across various cell input amounts.

Supplemental Note

**Note S1:** Standard Operating Procedure of π-SeqOmics

**Table S1**. Detailed parameters of the optimized experimental system for nucleic acid extraction and protein precipitation across various cell input amounts

|  | 5M | 1M | 500K | 100K | 50K | 10K |
| --- | --- | --- | --- | --- | --- | --- |
| Lysis Buffer | 350μL | 200μL | 100μL | 100μL | 50μL | 50μL |
| 70% Ethanol | 350μL | 200μL | 100μL | 100μL | 50μL | 50μL |
| Precipitant Buffer | 700μL | 600μL | 400μL | 400μL | 300μL | 300μL |
| Wash Buffer | 700μL | 600μL | 400μL | 400μL | 300μL | 300μL |
| Resuspension Buffer | 200μL | 100μL | 100μL | 100μL | 50μL | 50μL |

Supplemental Note 1. Standard Operating Procedure of π-SeqOmics

**(A) Reagents and Materials**

| Reagent name | Brand | Catalog number |
| --- | --- | --- |
| DNA/RNA co-extraction Kit | TIANGEN | DP422 |
| Zinc chloride(ZnCl_2_) | Sigma-Aldrich | 208086 |
| Sodium deoxycholate (SDC) | Sigma-Aldrich | D6750 |
| Tris(2-carboxyethyl)phosphine (TCEP) | Sigma-Aldrich | 646547 |
| 2-Chloroacetamide (CAA) | Sigma-Aldrich | C0267 |
| Tris(hydroxymethyl)aminomethane (Tris) | Pierce | 17926 |
| Trypsin, Gold, Mass Spectrometry Grade | Promega | V5111 |
| Fe-NTA Agarose | Cube Biotech | 31505-Fe |
| Methanol, LC/MS Grade | Fisher Scientific | A456-4 |
| Acetonitrile (ACN) , LC/MS Grade | Fisher Scientific | A955-4 |
| Formic acid (FA), LC/MS Grade | Fisher Scientific | 88315 |
| Trifluoroacetic acid (TFA) | Macklin | T875597 |
| Ammonium hydroxide (NH_4_OH, 25%) | Merck | 1054321011 |
| Absolute ethanol(EtOH) | Macklin | E809064 |

**(B) Reagent composition**

1. ZASP precipitation buffer: 200 mM ZnCl_2_, 99.9% MeOH, and 0.1%FA.

2. Washing buffer: 99.9% MeOH, 0.1%FA

3. 1%SDC Lysis: 1%SDC, 10 mM TCEP, 40 mM CAA, 100 mM Tris-HCl

4. Buffer A: 0.1%TFA

5. Buffer B: 80% ACN, 0.1%TFA

6. Fe-NTA loading buffer: 80% ACN, 0.1% TFA

7. Fe-NTA elution buffer: 60% ACN, 3% NH₃·H₂O

**(C) Detailed Experimental Procedure**

**Step 1: Sample Lysis**

1. Mouse tissues (10 – 20 mg) or HEK 293T cells (5 × 10^6^) were lysed using 350 μL RLplus lysis buffer. For varying sample inputs, reagent volumes throughout the workflow should be adjusted according to Table S1.

2. Mouse tissues were homogenized to ensure complete release of biomolecules.

**Step 2: DNA Isolation**

1. The lysate was transferred to a DNA Column and centrifuged (collect the flow-through for RNA extraction).

2. Add 500 μL buffer GD to wash the DNA column, centrifuge at 12,000 × g for 1 min.

3. Add 500 μL buffer PW to wash the DNA column, centrifuge at 12,000 × g for 1 min, and repeat this step once.

4. Add 100 μL of buffer TD to the DNA column and incubate for 2 min at 25°C.

5. Centrifuge at 12,000 × g for 1 min to elute DNA.

6. DNA was stored at -20°C.

**Step 3: Total RNA Isolation**

1. The flow-through from the DNA binding step was mixed with 70% ethanol and transferred to an RNA column, and centrifuged (collect the flow-through for protein extraction).

2. Add 700 μL Buffer RW1 to wash the RNA column, centrifuge at 12,000 × g for 1 min.

3. Add 500 μL Buffer RW to wash the RNA column, centrifuge at 12,000 × g for 1 min, and repeat this step once.

4. Add 100 μL RNase-Free ddH_2_O to the RNA column and incubate 2 min at 25°C.

5. Centrifuge at 12,000 × g for 1 min to elute RNA.

6. RNA was stored at -20°C.

**Step 4: Protein Recovery**

1. Add an equal volume of ZASP precipitation buffer to the flow-through from the RNA binding step, and incubate for 10 min at 25°C, and then centrifuge (19,000 × g, 10 min) to obtain the Protein pellets.

2. Add an equal volume of washing buffer to wash the Protein pellets and utilize ultrasonic-assisted cleaning (20s On, 20s Off, 85% energy), followed by centrifugation and air-drying.

**Step 5: Protein Resuspension, Reduction, and Alkylation**

1. Add 100 μL 1%SDC Lysis(0.1%SDC, 10 mM TCEP, 40 mM CAA, 100 mM Tris-HCI) to the protein pellets and utilizing ultrasonic-assisted resuspension.

2. Incubate the resuspension at 65°C for 10 min for reduction and alkylation.

**Step 6: Trypsin Digestion**

1. Add trypsin at a ratio of 25:1 (protein: trypsin), vortex, and then incubate at 37°C for 14 hours.

2. Terminate the enzymatic digestion by adding 1 μL of TFA. Vortex to mix, then centrifuge at 1,9000 × g for 10min; retain the supernatant.

**Step 7: C18 StageTips desalting**

1. Construct a StageTip by adding 6 mg of C18 powder and two layers of C18 membrane to a 200 μL tip.

2. Activate and equilibrate the peptide desalting column sequentially with 200 μL methanol, buffer B (80% ACN, 0.1% TFA), and buffer A(0.1% TFA), centrifuging at 3,000 × g at 25°C.

3. Load the supernatant into the StageTip and centrifuge at 3,000 × g; repeat this step once.

4. Wash the StageTip twice with 200 μL of buffer A by centrifuging at 3,000 × g.

5. Elute peptides with 50 μL of buffer B by centrifuging at 3,000 × g.

6. Dry the 10% peptide fraction in a SpeedVac concentrator and store at −80 °C until LC-MS analysis.

**Step 8: Phosphopeptides enrichment**

1. Construct a C8 membrane–based tip column by adding one layer of C8 membrane to a 200 μL tip.

2. Activate and equilibrate the C8 membrane–based tip column with 200 μL methanol and Fe-NTA loading buffer.

3. Add 10 μL Fe-NTA gel (at a ratio of 10 µL gel per 100 µg of peptides) to the remain 90% of the peptide solution and incubate with shaking for 30 min at 25°C.

4. Transfer phosphopeptdies enrichment solution to the C8 membrane–based tip column and 3,000 × g centrifugate at 25°C.

5. Wash the C8 membrane–based tip column twice with 200 μL of Fe-NTA loading buffer.

6. Elute phosphopeptides twice using 50 μL Fe-NTA elution buffer

7. Dry phosphopeptides in a SpeedVac concentrator and store at −80 °C until LC-MS analysis.
